# Worldwide Genomic Resources for Non-Model Fish Species

**DOI:** 10.1002/cfg.324

**Published:** 2003-10

**Authors:** Melody S. Clark, Douglas L. Crawford, Andrew Cossins

**Affiliations:** 1 HGMP Resource Centre Wellcome Genome Campus Hinxton, Cambridge CB10 1SB UK; 2 Rosenstiel School of Marine and Atmospheric Science University of Miami 4600 Rickenbacker Causeway Miami FL 33149-1098 USA; 3 School of Biological Sciences University of Liverpool The Biosciences Building Crown Street Liverpool L69 7ZB UK; 4 British Antarctic Survey High Cross Madingley Road Cambridge CB3 0ET UK


**Comparative and Functional Genomics**
*Comp Funct Genom* 2003; **4**: 502–508.
Published online in Wiley InterScience (www.interscience.wiley.com). **DOI:** 10.1002/cfg.324

**Feature**

### Meeting Review: Worldwide genomic resources for non-model fish species

Holmes Chapel, UK, 27–29 May 2003

Melody S. Clark[1#], Douglas L. Crawford[2] and Andrew Cossins[3*]
[1] *HGMP Resource Centre, Wellcome Genome Campus, Hinxton, Cambridge CB10 1SB, UK*
[2] *Rosenstiel School of Marine and Atmospheric Science, University of Miami, 4600 Rickenbacker Causeway, Miami, FL 33149-1098, USA*
[3] *School of Biological Sciences, University of Liverpool, The Biosciences Building, Crown Street, Liverpool L69 7ZB, UK*

**Correspondence to: Andrew Cossins, School of Biological Sciences, University of Liverpool, The Biosciences Building, Crown Street, Liverpool L69 7ZB, UK. E-mail: cossins@liv.ac.uk*

*[#] Current address: British Antarctic Survey, High Cross, Madingley Road, Cambridge CB3 0ET, UK.*

Received: 4 August 2003
Revised: 6 August 2003
Accepted: 6 August 2003

### Introduction

Fish genomics is developing an increasingly high profile, with the sequencing of four fish genomes (*Takifugu, Tetraodon*, zebrafish and medaka). However, numerous other fish species are used in laboratories throughout the world (the so-called 'alternative-model' or 'non-model' fish species), providing unique insights into fields ranging from physiology, toxicology and behaviour to evolution and ecology. The diversity in both species and experimental systems is in many ways both the strength and the weakness of fish as subjects for study. The strength comes from the sheer range of adapted forms and physiologies, many of which offer unique opportunities for exploring fundamental problems in biology. The weakness comes from a rather fragmented fish biology community, which is often species-centric, with collaborations restricted to a limited number of researchers working on the same (or similar) species or, in fewer instances, on the same phenomenon in diverse species. The aim of this discussion workshop was to bring together international fish scientists to review the current use of advanced genomic and post-genomic technologies in diverse fields of fish biology and to foster a new coherence in the coordinated development of screening technologies and the sharing of underpinning resources. This would not only invigorate gene function analysis but also enable new cross-taxon analysis of genome evolution.

The workshop was organized by **Andrew Cossins (Liverpool, UK)** and sponsored by the Natural Environment Research Council (NERC) Environmental Genomics Science Programme and the Biotechnology and Biological Research Council (BBSRC). The invited participants comprised an international mix of US, Japanese and European scientists, with representatives from the major

Copyright © 2003 John Wiley & Sons, Ltd.

UK funding bodies, the US National Science Foundation and industrial ecotoxicologists. Academic scientists from the major fish sequencing programmes were present alongside a cross-section of the more traditional disciplines, such as evolutionary and population biologists, ecotoxicologists, physiologists and developmental biologists.

### Fish as experimental models

Fish comprise almost half of all known vertebrate species (approximately 25 000 species), representing ∼500 million years of diversification. They survive in a diverse range of habitats from freshwater to hypersaline, −1.89 °C to ∼42°, oxygenated to completely anoxic, and present a huge range of naturally adapted forms and physiologies. Moreover, they are of great economic and nutritional importance through fisheries, with aquaculture contributing significantly to food production across the world. Finally, fish are critically important components of major ecosystems whose place is increasingly put at risk through overfishing. Fish diversity as a source of relevant experimental models was highlighted in this session with specific examples from neuroendocrinology, ecotoxicology and sexual differentiation.

**Richard Balment (University of Manchester, UK)** invoked Krogh's Principle, that for any problem there is a species best suited for its analysis. Within vertebrates, fish provide the diversity that allows the biologist to choose an appropriate fish species that best illustrates the issue under investigation. This often produces a clarity of approach and a view that is simply not evident when limited to mammals, enabling the biologist to access regulatory processes and decipher complex gene function. As an example of this, he cited his work on the control of body homeostasis. The euryhaline flounder is able move between freshwater and seawater habitats, a huge osmoregulatory challenge that induces profound ion regulatory responses in the principle osmoregulatory tissues. Whilst the flounder bears little physical resemblance to mammals, the ion regulatory transporters in its gill are homologous to those of the mammalian kidney tubule. Moreover, there are strong evolutionary links between the neurohumoral factors involved in osmoregulatory control in fish and mammals. Thus, exploration of gene and protein expression in flounder may have a direct bearing on, and facilitate the dissection of, gene function in mammals.

The role of fish as sentinels and subjects for ecotoxicology research was discussed by **Charles Tyler (University of Exeter, UK)**, particularly with respect to endocrine disruption. Being directly bathed by an aqueous environment, and interacting closely with it through their gills, they are directly and continuously exposed to water-borne chemicals. Whilst they cannot replace mammals as models for human toxicology, they offer important research tools to give advance warning of toxic effects, and for the prediction of the health implications and ecosystem level impacts of pollution. Moreover, some fish species, notably zebrafish and fathead minnow, offer technical advantages as models for ecotoxicological monitoring and post-genome screening.

**Laszlo Orban (TLL, Singapore)** discussed sexual differentiation in fish, a subject that is still very much a 'black box' due to the diversity of sexual systems evident fish (gonochorists, serial hermaphrodites, intersexes, etc.). Work in this area has a particularly important contribution to make in improving aquaculture and to the production of isogenic (clonal) lines. He made the important point that the fish whose genomes have been chosen for sequencing to date are not always the best models, as only one, medaka, has a defined sex chromosome system and proven, genetically-determined sex. It follows that understanding diversity in sexual differentiation and assignment requires exploration of other species.

Throughout this session it was made clear that no one fish species can satisfy all experimental requirements; physiologists prefer fish whose organs are large enough to manipulate, developmental biologists favour smaller species with rapid development times and transparent embryos, and yet others favour those with a clear link between behaviour or morphology and habitat, or with particular life history attributes. The diversity of fish presents many different opportunities to address specific scientific questions, and it is certain that the number of species under active investigation will continue to grow. Expanding the use of genomic techniques across this range, and the small communities involved, will make special demands upon scientists and funding agencies.

Copyright © 2003 John Wiley & Sons, Ltd.

### Phylogeny and trait reconstruction/evolution

The next session addressed components of fish phylogeny. **Michael Berenbrink (University of Liverpool, UK)** demonstrated how comparative physiologists can use phylogeny to reconstruct the evolution of complex physiological traits in vertebrates, using the examples of oxygen secretion in swim bladders and the choroid rete mirabilis of the eye. By mapping onto the phylogeny the sequential appearance of the key physiological, anatomical and molecular characters over time, he showed how complex integrated systems can evolve, giving insights into the dynamics and directions of evolutionary change. He demonstrated how these methods can also describe the evolution of genome size over 500 million years. Fish are particularly useful for phylogenetic reconstruction, since not only do they have a rich fossil record but some primitive taxa still have extant representative species ('fossil' fish), allowing characterization of living specimens.

This theme was continued by **John Postlethwait (University of Oregon, USA)** in his overview on gene duplication events in fish. It is well documented that fish have extra genes, which are thought to survive via the processes of non-functionalization, neo-functionalization and sub-functionalization. These gene duplications are seen by some as a drawback of fish models, but he showed that they can be very much an advantage. Using the zebrafish Sox 9 duplication as an example, he demonstrated how sub-function partitioning can break up pleiotropy. This provides an easier route to defining gene function, a situation that is not always possible in mammalian models (where the Sox 9 mutation is homozygous lethal).

### Genomic projects, mapping and ESTs

The rest of this day was devoted to genome projects, mapping and EST projects in both model and non-model species. This was probably the session that prompted the most discussion, particularly in relation to the most cost-effective strategy for extending the application of genomic techniques in fish biology.

Genome sequences are complete, or nearly completed, for zebrafish **(Kirsten Jekosch and Jane Rogers, Sanger Institute, UK)** and the puffer fishes *Takifugu* **(Greg Elgar, HGMP, UK)** and *Tetraodon*, and are being pursued in medaka **(Mitani and Shima, University of Tokyo, Japan)** and possibly in the Salmonidae. In addition, projects have been suggested for fathead minnow (US EPA), for stickleback and for three Antarctic nototheneid species (US, NSF Review of Polar Biology) and trout (NIH). EST collections are well under way for more than 20 species, some of which are linked to the production of microarrays **(Le Gac, Centre de Rennes, France)** and BAC libraries. From a phylogenetic viewpoint, the sequenced fish are concentrated within the Acanthopterygii (Figure 1). However attractive the initial proposal of sequencing representative fish species across the full phylogeny was to a largely academic audience, in the ensuing discussion this view was generally replaced by the consensus that fish biology would benefit more from heightened investment in related areas. These included greater international coordination of effort, production of an effective cross-species database federation, and in the wider development of techniques for experimental manipulation of gene expression. All experimental fish species are at different stages of genomic exploitation and it was felt that greater structuring of both current and future projects was required to give more collective power to the exploitation of fish genomics. Functional genomics investigations of new species can be quickly implemented through the generation of basic resources, particularly EST and BAC libraries. The latter can provide the platform for genome sequencing (via fingerprinting, end-sequencing and generation of markers), should the money for large-scale sequencing become available. However, in the interim, they also provide the ability to generate long-range continuity maps, and clones for sequencing of specific genes or regions of the genome.

A major problem that limits the applicability of sequence data to the full range of fish species is transferability of data between species; genetic maps are generally produced using species-specific microsatellite markers, so there is no link (and therefore no exploitation) possible between genetically mapped fish species or between genetic and physical maps. Whilst the *Takifugu* and zebrafish genome data are curated within Ensembl and comparative links between the two species are being

Copyright © 2003 John Wiley & Sons, Ltd.

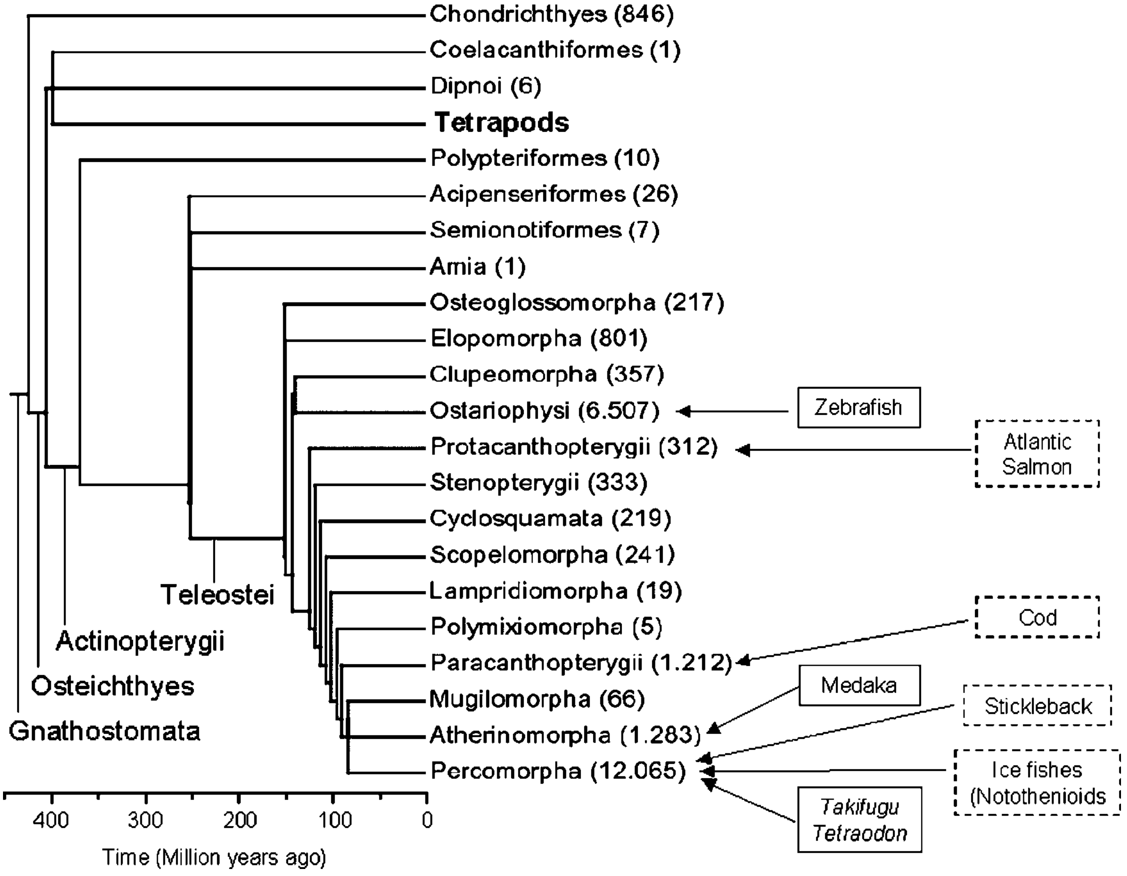

**Figure 1.** Phylogenetic tree of gnathostome fishes with the number of extant species given in brackets (after Nelson, 1994). Groups containing species with ongoing or prospective genome sequencing projects are indicated (solid and dashed lined boxes, respectively). The tree is calibrated according to the fossil record (based on Benton, 1993; Harland *et al.*, 1989)

developed, there is no current effort to extend this to other fish species. A strong message from the workshop was the requirement for a platform to facilitate comparative mapping between fish species with different types of markers and different scales of genomic information. An alternative would be the more widespread use of transferable markers (preferably using coding sequence) that, by facilitating positional cloning between species, would speed up forward genetic approaches in non-sequenced species. Radiation hybrid (**Robert Geisler, Max Planck Institute, Germany**) and HAPPY mapping (**Paul Dear, LMB, Cambridge, UK**) were proposed as alternative approaches to traditional genetic mapping that are ripe for exploitation using EST-generated markers.

The fragmentation of research interest across a wide range of different species and the evident lack of coordination between groups and programmes was continually revisited throughout the workshop, under all of the different areas of scientific investigation. Resources generated for the different fish species largely remain in the freezers of different laboratories. Indeed, there is no centralized resource centre, even within a single country, for the deposition, curation or dissemination of EST clone sets or BAC libraries. As a result, the utility and legacy of these expensive projects is likely to remain underexploited.

Another example was data analysis; many different groups have to deal with EST data annotation and are individually developing EST annotation pipelines and databases. The question of incompatible stand-alone databases was addressed by **Andy Law (Roslin Institute, UK)**. The essence of his talk was the need for database integration via federation using application programming interfaces (APIs). Database development is still relatively

Copyright © 2003 John Wiley & Sons, Ltd.

new in this area and it is essential to develop fully interactive relationships between different databases and to define vocabularies and external data references to provide the building blocks on which to re-engineer systems for increased integration of meta-data. The resulting overview of chromosomal organization and synteny, and the wider availability of sequence and clone resources would facilitate the more efficient exploitation of an even wider range of species.

### Comparative mapping

This session was opened by **Alan Teale (University of Stirling, UK)**, who gave a comprehensive overview of QTL analysis. He pointed out that much research is candidate gene-driven, but that knowledge of QTLs is required for a complete understanding of genotype–phenotype and gene–environment interactions. Detection and mapping of QTLs does not require prior knowledge of genes and their functions and goes directly to the genetic factors contributing to phenotypic variation. QTLs are not necessarily differentially expressed and therefore not all will be identified by a microarray approach. However, QTL analysis is demanding of time and resources and in many ways has been overlooked in the drive for genomics approaches.

The remainder of the session presented genetic analyses of adaptive traits in wild species. **David Kingsley (Stanford University, USA)** showed how pelvic reduction and patterning of armour plates in the stickleback can be approached using traditional positional cloning methods. Remarkably, each trait is controlled by a single major locus. **Tom Kocher (University of New Hampshire, USA)** explained how comparative approaches can be used to map genes underlying colour patterns and trophic morphology (jaws and teeth) of cichlid fishes. Markers flanking the QTLs for these traits were used to screen BAC libraries, and the corresponding clones partially sequenced to identify homologous regions of the *Takifugu* scaffolds and the medaka map. Conservation of synteny was demonstrated over intervals of 30 cM, and the *Takifugu* sequence data was used to generate additional markers in the interval. These studies showed the feasibility of identifying the genetic basis of natural variation associated with speciation.

### Functional genomics

The main focus of this session was on the use of microarray technology, with talks from **Douglas Crawford (University of Miami, USA), Andrew Cossins (University of Liverpool, UK)** and **Thomas Dickmeis (IGBMC, France)**. They showed how microarrays can be used to analyse cardiac metabolism in *Fundulus*, responses to cold in carp, and axial midline development in zebrafish, respectively. In all cases, analysis of large microarray sets of 15–18 000 genes yielded a much smaller, defined sub-set of genes, which could then be subjected to further analysis. In a variation on the 'usual' microarray talk, Crawford showed how the technology could be used to answer questions surrounding the importance of variation in transcript expression. Using cardiac metabolism in *Fundulus*, he showed that expression levels of particular genes vary within (18% of loci were significantly different) and between populations. After a series of microarray experiments, the question then arises of 'what to do with the list of genes?'. Due to the lack of knock-out technology in the carp, Andrew Cossins' group investigated candidate genes using RNAi in *C. elegans*. Thomas Dickmeis used *Takifugu*/zebrafish genomic comparisons to identify conserved promoter and enhancer elements in his candidate genes, which were then tested for their spectrum of expression in zebrafish transgenics.

This session also provoked considerable debate, over the technologies themselves but also the potential of microarrays for cross-species use. This would greatly expand the utility of such expensively constructed resources, but is likely to depend on whether the arrayed probes are cDNAs or oligos. Oligos to untranslated regions offer greater discrimination between isoforms or members of a gene family but might not favour cross-species use. Judicious selection of oligos in both conserved translated and non-conserved untranslated regions might resolve this apparent trade-off.

### Transgenics and genome manipulation

The final session concentrated on genome manipulation. **Hans Komen (Wageningen Universiteit, The Netherlands)** gave an overview of the 'traditional' approaches for producing isogenic lines,

Copyright © 2003 John Wiley & Sons, Ltd.

which are powerful tools for genetic analysis. Surprisingly, there has been very little improvement in the methodology over the past 20 years; heat shock and pressure are still used, and can produce phenotypic variation. Efficiencies are still low, at 2–8% of treated eggs, and few lines are available, mainly in trout, tilapia and carp. The most successful species are members of the Salmonidae and Cyprinidae, perhaps because these groups have polyploid members.

**Norman Maclean (University of Southampton, UK)** then gave an overview of current transgenic technologies. These provide a means of exploring in detail the time and place of expression of individual gene promoters, enhancers and other regulatory sequences, particularly in developing embryos. However, these methods are slow and labour-intensive and only deliver assessment of candidate genes, rather than a high-throughput screening method. Fish transgenesis is still mainly performed by microinjection into the perinuclear cytoplasm of newly fertilized eggs, although electroporation, sperm-mediated gene transfer, liposome-mediated gene transfer and gene guns offer alternatives. Expression of transgenes in embryos is typically transient and mosaic, although in rare cases they do integrate and individuals expressing in subsequent generations can be identified using suitable markers. Integration of transgenes can demonstrably produce fish with genetically manipulated characteristics for either experimental or aquacultural purposes, although again the methodology is difficult and specialized. Fish of many species have been made transgenic, including trout, salmon, catfish, carp, tilapia, stickleback, zebrafish and medaka. Gene knockdown with antisense has been demonstrated but, so far, RNAi does not seem to work in fish.

**Manfred Schartl (Universitat Wurzberg, Germany)** expanded upon the previous talk, discussing the development of fish ES cell lines. Success so far has been very limited. What is becoming clear is that successful ES cell lines are limited to certain host/donor genotypes and that these influence the amount of chimaerism. Homologous recombination is another possible technology, but data in fish is limited and it is not routinely usable (with success rates of 1/4000 in medaka). Nuclear transfer is a more distinct possibility and has been carried out in cyprinids, medaka, zebrafish and loach. However, this technology is very species-specific and the technological expertise is limited to specialist labs. Clearly, these are emerging technologies that offer great potential for the future, but still require a lot of development work.

### Conclusions

The strength of fish genomics is the incredible diversity of the group. Importantly, interesting biological differences occur among closely related, and thus more recently diverged taxa, although they can also be traced back ~500 million years using extant representatives of ancient forms. Thus, fish can illuminate questions of recent, as well as long-standing, adaptation of phenotypes, and a major challenge is to explore this in relation to the evolution of the genome. Investigators can examine, for example, differences in thermal phenotype between cyprinid and salmonid fish over 100 million years, metabolic differences in heart function among *Fundulus* species that have diverged less than 10 million years ago, developmental changes in the jaw morphology among cichlid species that have diverged less than 1 million years ago, and ecological, morphological and behavioural changes among sticklebacks in the last 10 000 years. Fish can act as valuable models for studying a range of fundamental questions, from physiology to ecology and evolution, and they can even have a direct bearing on questions of biomedical significance. This was evident in identifying genes coding for skeletal or morphological variation in sticklebacks and cichlids, for transporter identification in osmoregulatory tissues and in the effective dissection of pleiotropic promoters of mammals. Moreover, fish can offer technical advantages over other vertebrate systems, beyond those recognized in zebrafish and medaka, although the lack of high-throughput techniques for gene manipulation remains an impediment in most species, at least in relation to what can now be achieved in *C. elegans*.

Currently there is too little integration of fish genomics programmes and the benefits of critical mass will not be realized until the means are available to seamlessly relate expression and sequence data between species. Linking the physical and genetic maps of non-sequenced species to the finished genome sequences of the few genomic models will allow the significant investment made in the genome sequences to become of benefit to

Copyright © 2003 John Wiley & Sons, Ltd.

the broader community. This requires an improved coordination of effort to make genomic resources more widely available, but also a need to unify databases, to define relevant and controlled vocabularies, to link external data references and to provide training to ensure compliance. These will provide the informatic building blocks on which data from the whole community can be integrated and exploited. It will also facilitate a paradigm shift from the application of genomic science as a high-throughput data generating toolbox to a more focused knowledge-based approach to addressing biological problems.

These coordination activities represent a particular challenge, given the range of fish species under investigation. In addition, research activities are funded via a number of different international funding bodies with diverse scientific agendas, and there are diverse requirements for data and resource curation and management within the community. Moreover, depending on circumstances, research funding for each species might need to be justified on the basis of scientific outcomes for that species, rather than for reasons of phylogenetic inclusion or correctness. Nevertheless, the resulting meta-analysis, taking phylogenetic position into account, is likely to underpin a whole new understanding of the mechanisms and dynamics of genome evolution and of comparative genomics. It is important that funding agencies and stakeholders recognize the added value of participating in the grander scheme. To give an example, whilst the academic fish ecotoxicologist might explore dose–response curves for the fathead minnow, stakeholders (industry and regulators) would be interested in ways of improving the scientific basis for extrapolation from sentinel fish species to others. Therefore, the onus is now on the national and supranational funding agencies to engage with their science communities, and with each other, to realize the potential for the effective integration of genomic science in fish biology.

### Acknowledgements

This workshop was funded by the UK National Environmental Research Council (NERC), under their Environmental Genomics Thematic Programme and also The Biotechnology and Biological Sciences Research Council (UK). We would also like to thank Tom Kocher, Norman Maclean, Alan Teale, Florence Le Gac and Jason Snape for their contributions to the manuscript, Jo Wixon for critical reading of the manuscript and Michael Berenbrink for Figure 1.

### References

1. Benton MJ. 1993. *The Fossil Record*, vol 2. Chapman & Hall: London.
2. Harland WB, Armstrong RL, Cox AV, Craig LE, Smith AG, Smith DG. 1990. *A Geologic Time Scale 1989*. Cambridge University Press: Cambridge.
3. Nelson JS. 1994. *Fishes of the World.* Wiley: New York.

Copyright © 2003 John Wiley & Sons, Ltd. 

